# Supplementation of rumen-protected choline during warm summer days reduces body surface temperature and improves dry matter intake of Holstein dairy cows

**DOI:** 10.3168/jdsc.2025-1000

**Published:** 2026-05-22

**Authors:** I. Abousoliman, J. Schwerdtfeger, F. Koch, M.R. Schneider, B. Kuhla

**Affiliations:** 1 Research Institute for Farm Animal Biology (FBN), Dummerstorf, 18196, Germany; 2 Institute of Veterinary Physiology, Leipzig University, Leipzig, 04103, Germany

## Abstract

Elevated ambient temperature negatively affects dairy cows' productivity. The present study investigated the efficacy of rumen-protected choline (RPC) supplementation in alleviating the adverse effects of heat load and improving the productive performance in multiparous Holstein dairy cows at elevated ambient temperatures. Two multiparous cow groups, each enrolled since preceding calving, underwent a 2-phase experimental design consisting of a 1-wk pre-experimental period (wk −1) and a 4-wk experimental period (wk 1–4) in the same free-ranging barn. At the start of the pre-experimental period, cow groups were randomly assigned to a control group (CON, n = 9, 77 ± 26 DIM) receiving a basal diet throughout the study, whereas the RPC group (RPC, n = 9, 77 ± 8 DIM) received the same basal diet during the pre-experimental phase and was supplemented with RPC (60 g/cow per d) during the experimental phase. During the study in July 2025, the weekly mean ambient temperature ranged from 18.3°C ± 1.2°C to 20.9°C ± 1.8°C, with corresponding temperature-humidity index (THI) values between 64.1 ± 1.7 and 67.9 ± 2.6. Respiration rate was measured during the pre-experimental period (wk −1, d −6). Rectal temperature was determined using a digital thermometer and surface temperatures (muzzle, ear, eye, and neck) by aFLIR T620 infrared camera, during both the pre-experimental (wk −1, d −6) and experimental (wk 3, d 20) phases. Additionally, DMI, water intake (WI), milk yield (MY), ECM, BW, and milk composition were recorded for each cow individually in both periods. No significant differences were detected between the 2 groups during the pre-experimental phase, except for DMI, WI, BCS, and milk lactose percentage; however, pre-experimental differences were accounted for in the model. During the experimental period, RPC supplementation reduced eye temperature (RPC 34.7°C ± 0.1°C vs. CON 35.1°C ± 0.1°C) and tended to reduce ear (33.6°C ± 0.2°C vs. 34.4°C ± 0.4°C), muzzle (33.0°C ± 0.3°C vs. 33.6°C ± 0.2°C), and neck (34.3°C ± 0.2°C vs. 34.8 ± 0.2°C) temperatures. Moreover, RPC supplementation increased DMI (22.5 ± 0.47 vs. 18.6 ± 0.47 kg/d), WI (95.3 ± 1.05 vs. 71.6 ± 0.72 L/d), and BW (676 ± 3.53 vs. 657 ± 2.38 kg) relative to the CON group. Furthermore, RPC supplementation significantly increased milk fat yield (1.24 ± 0.04 vs. 1.16 ± 0.04 kg/d). Overall, the findings show that RPC supplementation may serve as an effective nutritional intervention to alleviate the negative effects of high ambient temperatures and to improve productivity in dairy cows.

## Highlights

• Heat-stressed cows fed RPC showed lower body surface temperatures.
• RPC increased the milk fat yield of heat-stressed dairy cows.
• RPC may serve as a supplement facilitating adaptation to high temperatures.

## Graphical Abstract

**Figure:**
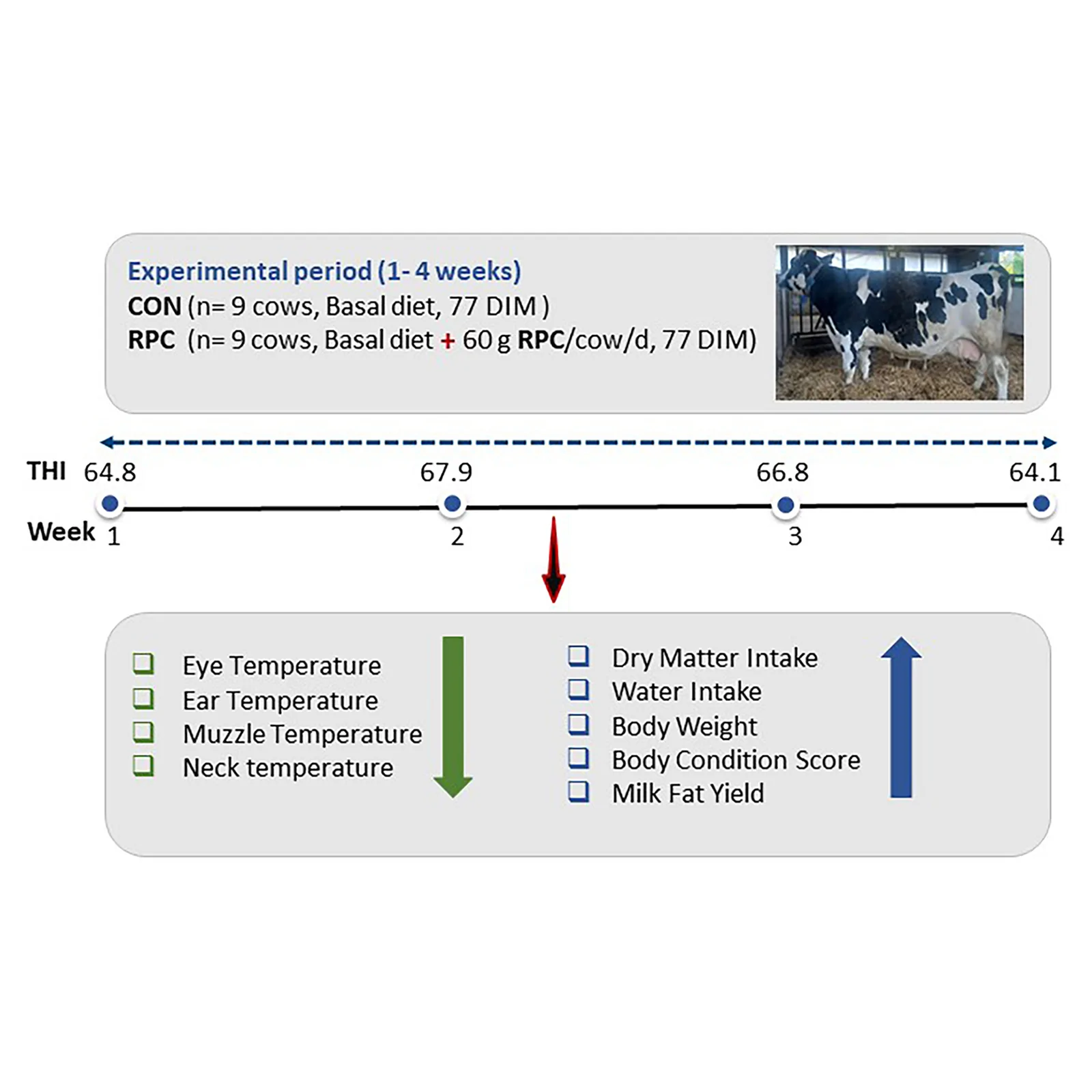
**Summary:** The primary objective of this study was to assess the efficacy of rumen-protected choline (RPC) supplementation in mitigating the adverse effects of heat load at elevated ambient temperatures in Holstein dairy cows. The impact of RPC on productive performance, including dry matter intake (DMI), water intake (WI), energycorrected milk, body weight (BW), body condition score (BCS), milk yield, and milk composition, was also investigated. Supplementation with RPC resulted in a significant effect on eye temperature reduction and a trend toward lower muzzle, ear, and neck temperatures. In addition, DMI, WI, BW, BCS, and yield of milk fat were significantly increased by RPC supplementation. These results indicate the potential of RPC supplementation as a nutritional strategy to alleviate the detrimental impacts of heat stress on dairy cows. CON = control; THI = temperature-humidity index.

Climate change has recently led to an increase in ambient temperatures worldwide, particularly during the summer months in which heat waves occurred more frequently. Elevated temperatures negatively affect dairy cattle productivity by disrupting various biological processes and compromising the overall well-being of the animals. These effects include direct impacts on physiological responses, such as increased respiration rate and body temperature (Koch et al., 2023). In addition, heat stress reduces feed intake, which subsequently leads to a decline in milk production and milk quality (Al-Qaisi et al., 2019). When the daily mean temperature-humidity index (**THI**) in Western European barns increases above 56 to 60, dairy Holstein cows display first signs of heat stress, including alterations in behavioral patterns such as lying and standing time (Hut et al., 2022). A study conducted in Germany between 2003 and 2022 reported that number of heat stress days increased by one per year, reaching up to 120 d annually in some regions. During summer months (June to August), average environmental conditions of 18°C, 72% relative humidity, and THI of 64–65 were observed, which were associated with an approximately 10% reduction in milk yield (**MY**; Leandro et al., 2024). To mitigate the adverse effects of high THI, feed additives such as vitamins, minerals, prebiotics, probiotics, yeasts, essential oils, herbs, enzymes, and choline have been explored for their potential to maintain normo-physiological conditions in dairy cows (Holdorf and White, 2021; Kotsampasi et al., 2024).

Choline is a precursor for phosphatidyl choline synthesis supporting very-low-density lipoprotein (**VLDL**) formation and thus the export of long-chain fatty acids, the latter diminishing the development of fatty liver during the transition period of dairy cows (Arshad and Santos, 2024; Ghaffari et al., 2025). Additionally, choline is involved in betaine synthesis, which supports energy metabolism (Supriyati et al., 2016) and also has a role in oxidative stress defense and immune function (Garcia et al., 2018). The combination of these actions relieved metabolic load and improved milk production and milk quality of early-lactating dairy cows (Mečionytė et al., 2022), goats (Supriyati et al., 2016; Habeeb et al., 2017), and sheep (Crosby-Galvan et al., 2024) supplemented with rumen-protected choline (**RPC**). Choline further serves as building block for the neurotransmitter acetyl choline, which, among others, promotes cutaneous vasodilation, blood flow, and sweat production (Hu et al., 2018; Stiegler et al., 2021), all of which potentially facilitate heat dissipation via the skin. Recently, it was shown that RPC supplementation of Holstein dairy cows wearing an electric heat blanket reduced respiration rate (Holdorf and White, 2021), and lower rectal temperatures were reported in early-lactating cows supplemented with RPC compared with nonsupplemented counterparts (Zenobi et al., 2020). Thus, the inclusion of RPC into the diet of ruminants seems to be a promising nutritional strategy to mitigate negative effects of heat stress. However, the mechanisms facilitating heat dissipation via the skin and mitigating heat stress during RPC feeding at high ambient temperatures are not deeply investigated yet. We aimed to prove the hypothesis if RPC supplementation reduces the body surface temperature and increases the productive performance in Holstein dairy cows during hot summer weeks.

The study was conducted in the free-ranging barn of the Research Institute for Farm Animal Biology (FBN) during summer 2025 (July 1 to August 2). The animal experiment was approved by the Landesamt für Landwirtschaft, Lebensmittelsicherheit und Fischerei Mecklenburg-Vorpommern (LALLF), Germany. The present feed trial required no extra permission. The barn was equipped with climate sensors placed at a height of 1.5 m above the floor. Ambient temperature (Ta) and relative humidity (RH) were recorded by the respective sensors (better Networks, Großharthau-Bühlau, Germany) every 20 min. Wind speed (Ws) was recorded every 5 min using wind transmitters (Fuehler Systeme eNET, Nürnberg, Germany). Solar radiation (Sr) was also measured every 5 min using Pyranometers (LPPYRA03, Delta Ohm, Remscheid, Germany). The THI and Comprehensive Climate Index (**CCI**) were calculated according to Mader et al. (2010) and Yan et al. (2021) as follows:

THI = (1.8Ta + 32) − (0.55 − 0.0055RH) × (1.8Ta − 26),

CCI = Ta + Eq.(RH) + Eq.(Ws) + Eq.(Sr),

where Eq. = respective equation for RH, Ws, and Sr by Mader et al. (2010). In total, 18 multiparous Holstein cows were kept in 2 pens, with dimensions of 22.5 m × 11 m for each pen. Each pen was equipped with 10 feeders and 2 water bins but not with fans and soakers. Nine socially integrated cows were allocated to each pen. One cow group was randomly assigned as the control (**CON**; n = 9; mean ± SE: 30.36 ± 0.35 kg/d MY; 77 ± 26 DIM; 660 ± 3 kg BW), while the second group received RPC supplementation (RPC; n = 9; 33.90 ± 0.24 kg/d MY; 77 ± 8 DIM; 674 ± 4 kg BW). Cow groups were subjected to a 2-phase experimental design consisting of a 1-wk pre-experimental period (wk −1) and a 4-wk experimental period (wk 1 to 4). In the pre-experimental period, all cows were fed the same TMR for ad libitum intake. In the 4-wk experimental period, the CON group continued to be fed the TMR. Cows in the RPC group were fed the same diet as the CON group but supplemented with 60 g RPC/cow per day (ReaShure-XC, Balchem, NJ), which was mixed with the TMR. The amount of RPC supplementation was chosen from the study by Holdorf and White (2021). The TMR on a DM basis consisted of corn silage (279 g/kg), meadow grass silage (367 g/kg), concentrate (348 g/kg), and mineral feed (6 g/kg) and had a ME content of 11.0 MJ/kg. Concentrate consisted of corn meal (22.6%), rapeseed extraction meal (26.7%), faba bean meal (12%), lupin meal (12%), corn gluten (7.8%), wheat (13.2%), mineral mixture (2%), feed lime (1.3%), and soy oil (0.8%). Cows were fed at 0430 and 1430 h. The respiration rate was measured by counting flank movements over a 15-s interval and multiplying the value by 4 to obtain breaths per minute, measured between 1400 and 1500 h on d −6 of the pre-experimental period. Rectal temperature in an insertion depth of ∼10 cm was determined using a digital thermometer (veterinary thermometer SC 1080, SCALA Electronic GmbH, Stahnsdorf, Germany). Body surface temperatures including muzzle, ear, eye, and neck temperatures were measured using an infrared camera (FLIR T620, Teledyne FLIR, Teledyne FLIR OEM, Belgium). Images were obtained only once during the pre-experimental period (d −6) and once on d 20 (experimental period) due to limited camera availability. The images were taken for each cow individually in different positions in range of 1.5 to 2 m far from the cow inside the barn without restraining the animals. One image from each cow that clearly showed the body surface was selected for analysis. The infrared images were analyzed using FLIR thermal studio software (Teledyne FLIR, Belgium). Feed intake was measured daily for each cow individually using Insentec feeders and water intake (**WI**) by Insentec water-weigh troughs (Insentec RIC, Marknesse, the Netherlands). Weekly feed samples were collected to determine the DM content, which allowed the calculation of DMI for each cow. The DMI for each group was calculated for 24 h and for the photoperiod length: daytime (9 h, 0900 to 1800 h) and nighttime (15 h, 1800 to 0900 h). Milk yield was automatically recorded from an Automated Milking System equipped with a milk meter (DeLaval GmbH, Glinde, Germany) for each cow at each of the 2 daily milkings (0500 and 1630 h) throughout the experiment. Milk samples were obtained weekly as a pool for each cow from the afternoon and next morning milking. Milk samples were preserved using Bronopol (Schmehl Laborausstattung, Rostock, Germany) and sent to the Milchkontroll and Rinderzuchtverband eG (Güstrow, Germany) for the analysis of fat, protein, and lactose by mid-infrared spectroscopy (MilkoScan, Foss GmbH, Rellingen, Germany). The weekly component yield was calculated by multiplying the respective milk composition and yield. The ECM yield was calculated from milk composition and milk yield (**MY**) according to following equation (Koch et al., 2024): ECM (kg/d) = [0.038 × fat (g) + 0.024 × protein (g) + 0.017 × lactose (g)] × MY (kg/d)/3.14. Body weight was measured after each milking using a walk-through scale and BCS was recorded daily by DeLaval BCS camera (DeLaval, Glinde, Germany).

Daily repeated measurements on the same animal were used to calculate the weekly means, which were analyzed by repeated measurement ANOVA using SPSS V20 (IBM, New York, NY). In the pre-experimental period (wk −1), the statistical model included the fixed effect of group (CON and RPC) and lactation number as covariates. For the experimental period (wk 1– 4), the model included group (CON vs. RPC), time (n = 4), and the group × time interaction as fixed effects; animal ID as a random effect; and lactation number and the baseline value at wk −1 as covariates. For respiration rate, and rectal and surface temperatures, the fixed effect was group (CON and RPC). Residuals were tested for normality and homogeneity using the Shapiro–Wilk test. Weekly means and their standard errors were computed for each fixed effect in the model. Additionally, differences of the means were tested using the independent samples *t*-test. Significance was declared at *P* ≤ 0.05 and a tendency when 0.05 < *P* ≤ 0.1.

The weekly mean values for ambient temperature, relative humidity, THI, and CCI recorded during the experiment are presented in Figure 1. In the pre-experimental phase, the weekly mean ambient temperature, relative humidity, THI, and CCI were 20.8°C ± 3.0°C, 63% ± 6.5%, 67.0 ± 3.7, and 23.9°C ± 3.6°C, respectively. During the experimental phase, the weekly mean ambient temperatures ranged between 18.3°C ± 1.2°C and 20.9°C ± 1.8°C, whereas relative humidity varied between 73.4% ± 5.1% and 81.7% ± 4.8%. The THI values ranged from 64.1 ± 1.7 to 67.9 ± 2.6, and CCI from 22.2°C ± 1.8°C to 26.1°C ± 2.2°C. During the pre-experimental period, THI was 68.5 during the daytime and 65.1 at nighttime, whereas in the experimental period, the THI values were 67.4 and 64.4 for daytime and nighttime, respectively. The mean respiration rate of all cows recorded on d −6 was 77.0 ± 5.4 breaths/min (THI = 74.6) and did not differ between cow groups. This respiration rate exceeded the threshold of 60 breaths/min defining onset of heat stress in lactating dairy cows (Toledo et al., 2020). However, the respiratory rate determined in the present study corresponds to previous counts of 71.8 breaths per minute of dairy cows experiencing heat stress induced by an electric heat blanket for 7 d (Holdorf and White, 2021). Unfortunately, we could not measure respiration rate during the experimental period, but a recent study showed that RPC supplementation (60 g/cow per d) tended to reduce respiration rate of heat-stressed Holstein dairy cows (Holdorf and White, 2021).

**Figure 1:**
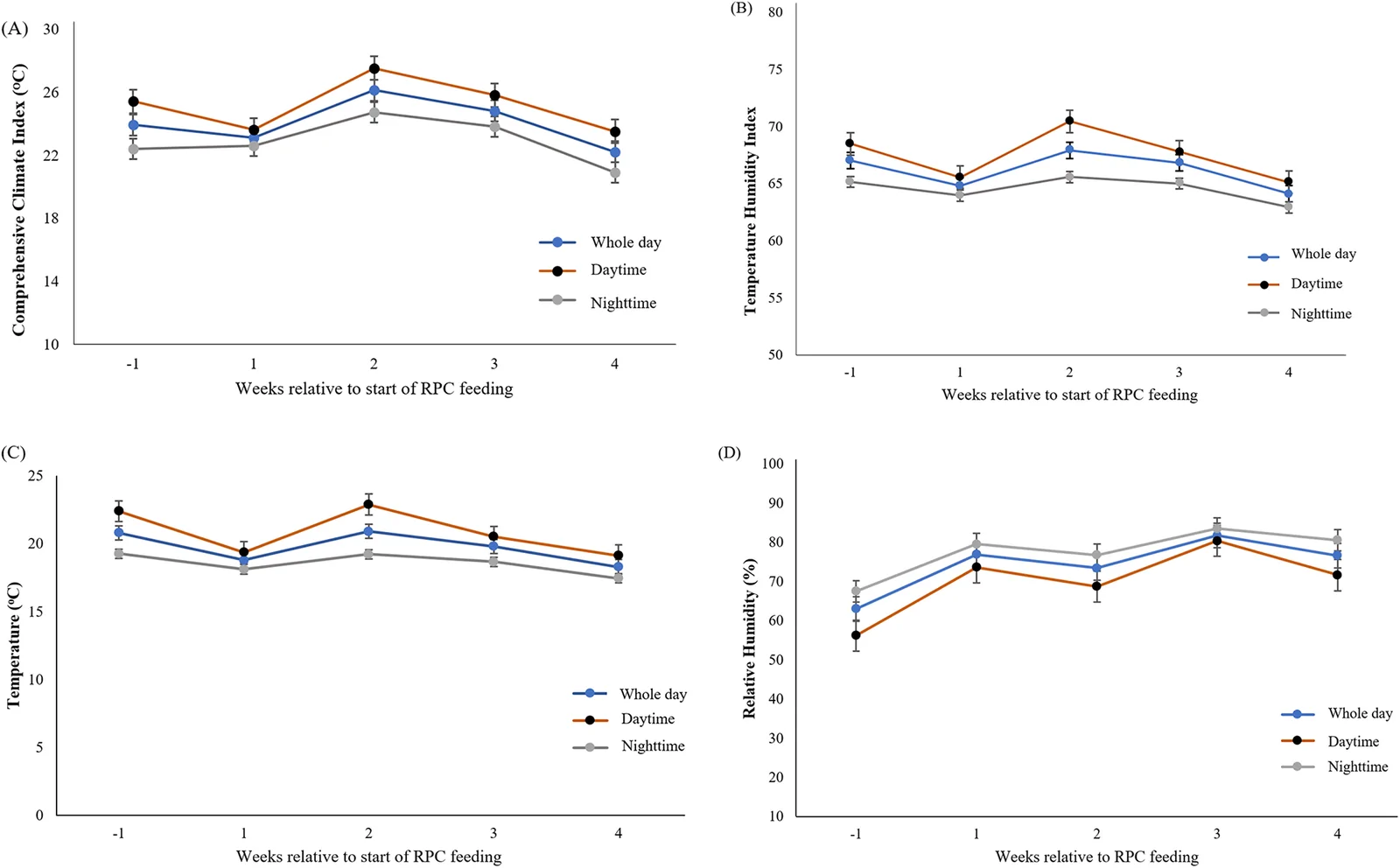
Weekly variation of the average Comprehensive Climate Index (A), temperature-humidity index (B), ambient temperature (C), and relative humidity (D) during the pre-experimental period (wk −1) and the experimental period (wk 1 to 4). For each parameter, the average of the whole day (0000 to 2400 h, blue), daytime (0900 to 1800 h, orange), and nighttime (1800 to 0900 h, gray) is illustrated. All data presented as mean ± SE.

Rectal temperature as well as the surface temperature of the muzzle, ear, and eye did not differ between cow groups in the pre-experimental period (*P* > 0.1). In contrast, in wk 3 of the experimental period, the eye temperature of RPC cows (34.7°C ± 0.11°C) was significantly lower (*P* = 0.05) than in the CON group (35.1°C ± 0.14°C; Figure 2). The RPC-fed group tended to have lower ear (33.6°C ± 0.24°C vs. 34.4°C ± 0.35°C; *P* = 0.08), muzzle (33.0°C ± 0.26°C vs. 33.6°C ± 0.23°C; *P* = 0.10), and neck temperature (34.3°C ± 0.18°C vs. 34.8°C ± 0.20°C, *P* = 0.09), suggesting that RPC supplementation reduced heat load by facilitating heat dissipation from the body surface. It has been shown that increased intake of choline increased tissue acetyl choline concentrations (Cohen and Wurtman, 1976), and central choline injection induced hypothermia in rats (Unal et al., 1998). Furthermore, cutaneous choline injection (Yue et al., 2020) and increased choline ingestion facilitated sweating in humans (Alkan and Atakan, 2011). These results highlight the mechanisms underlying the reduced surface temperatures of RPC-fed cows, potentially including increased sweating and heat dissipation. However, rectal temperature was not lower during RPC feeding (Figure 2), and this finding corresponds to earlier studies reporting no change in rectal temperature after feeding RPC to dairy cows (Holdorf and White, 2021) and dairy goats (Habeeb et al., 2017) experiencing heat stress. Giving that body temperatures may vary during the day and over time, the low number of surface and rectal temperature measurements is a limitation of our study that may have influenced the results.

**Figure 2:**
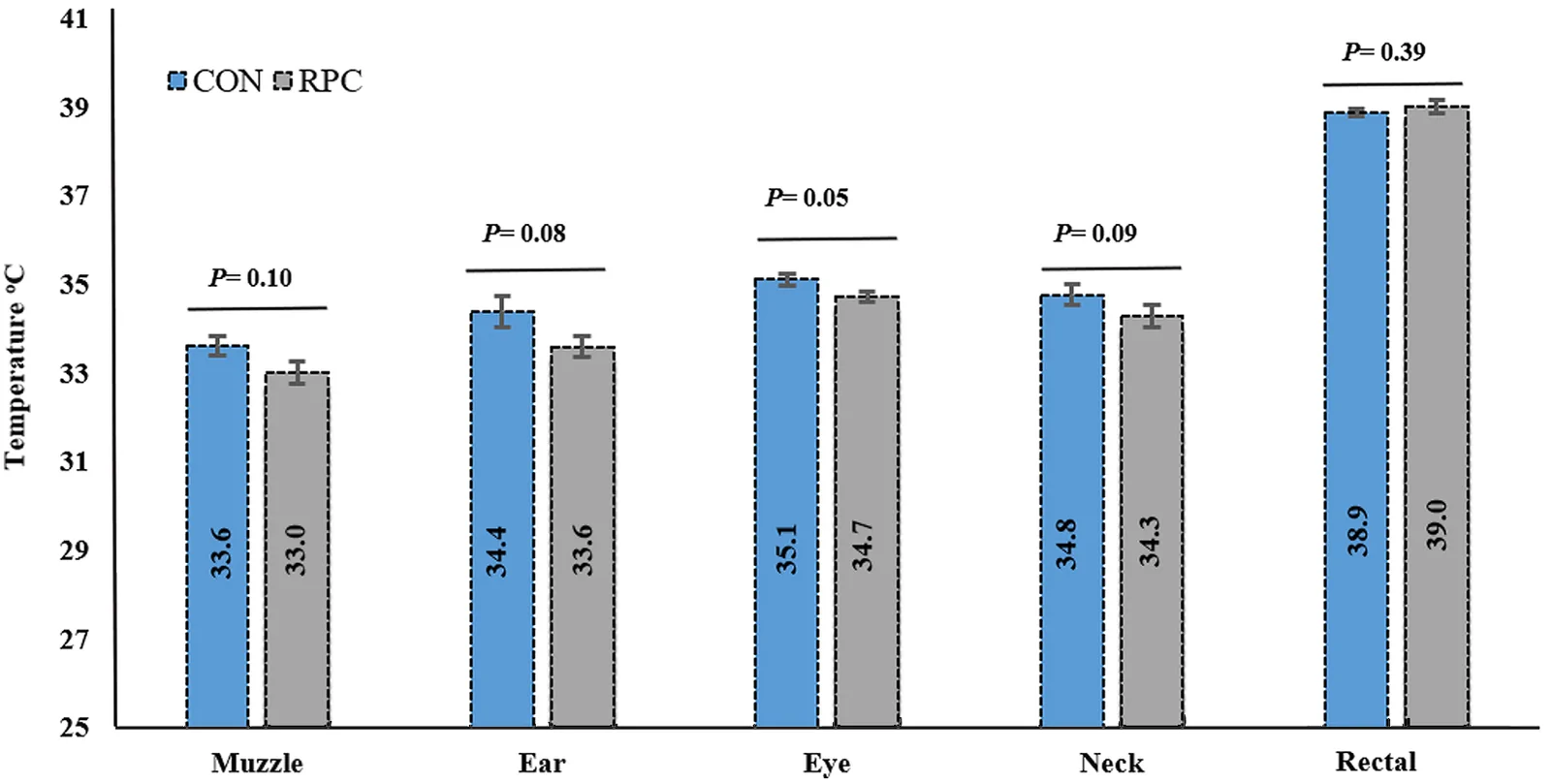
Body surface temperatures (muzzle, ear, eye, neck) measured by an infrared camera and rectal temperature measured by a digital thermometer of RPC-fed and control (CON) cows in wk 3 (d 20) of the experimental period. All data are presented as mean ± SE, n = 9 cows per group.

Table 1 shows the daily DMI and WI of the RPC and CON groups. Both cow groups differed in these variables already in wk −1 of the pre-experimental phase, primarily because of the lower DMI during the daytime but not nighttime of the CON group. However, wk −1 measurements were included as a covariate in the statistical analysis of the experimental period to account for the pre-existing differences. The greater increase in DMI after the start of RPC feeding is potentially owed to a higher heat tolerance achieved by increased heat dissipation. Similarly, technical approaches facilitating heat dissipation (e.g., the use of evaporative adiabatic cooling combined with forced ventilation systems) have been shown to maintain body temperature and DMI at high ambient temperatures (Oliveira et al., 2025). During the experimental period, RPC-fed cows had a higher BW than the CON group (*P* < 0.01; Table 1), indicating that the RPC supplementation supported BW gain during the last 4 wk of the trial. The greater BW of the RPC group can be partially explained by their higher DMI during the 4-wk experimental period. Indeed, DMI normalized to BW was also greater in RPC than CON cows (0.031 vs. 0.026) in the experimental period (*P* < 0.01; Table 1). The BW gain of RPC-supplemented cows is reflected by the increase in BCS of RPC cows and the decreasing BCS difference between groups from the pre-experimental (ΔBCS: 0.23) to the experimental period (ΔBCS: 0.18). No significant differences in MY between RPC and CON group were noticed (*P* = 0.76; Table 1). Similarly, ECM was not different between the control and treatment groups (*P* = 0.18). Milk constituents did also not differ between groups (*P* > 0.05) in the experimental phase, except milk fat yield, which was significantly higher (*P* = 0.05) in RPC than CON cows (Table 1). No effect was reported for RPC (6 to 50 g/d) feeding on milk fat content of cows being 0 to 176 DIM (Sales et al., 2010). The increase in milk fat yield observed in our study may be attributed to choline's role as precursor for the synthesis of phosphatidyl choline, the latter facilitating the transport of mobilized free fatty acids and triglycerides from adipose tissue and liver to the mammary gland, thereby improving liver function and enhancing the availability of fatty acids for milk fat synthesis (Brusemeister and Sudekum, 2006; Pinotti et al., 2008; Supriyati et al., 2016). Two recently conducted meta-analysis studies showed that supplementation of 13 to 24 g RPC ions per day to the diet of prepartum and early-lactating cows increased milk protein yield (Arshad et al., 2020; Ghaffari et al., 2025). Choline's function as a methyl donor may spare S-adenosylmethionine, which is important for maintaining energy metabolism as well as for methionine synthesis, the limiting AA for milk protein production (Sales et al., 2010). However, none of these studies reported the climate conditions the cows were exposed during RPC supplementation. Thus, it seems that RPC supplementation does not facilitate milk protein synthesis during heat stress. Holdorf and White (2021) studied the impact of RPC feeding in dairy cows subjected to heat stress for 7 d using an electric heat blanket during the late lactation period. Despite 60 g/d RPC supplementation, the heat-stressed cows responded by a reduction in DMI (1.2 kg/d), ECM yield (3.2 kg/d), milk fat yield (0.12 kg/d), milk protein yield (0.1 kg/d), and milk lactose yield (0.15 kg/d) to the same extent as the control group (Holdorf and White, 2021). These findings agree with our assumption that RPC has no major impact on milk protein yield at higher ambient temperatures. However, the decline in MY, DMI, and milk fat yield in the Holdorf and White (2021) study is in contrast to our results but may be explained by the fact that the heat blanket tied around the abdomen of the cow prevented heat dissipation via the skin and thus RPC's action on reducing skin temperature. Another reason may be the progressing stage of lactation, during which milk production naturally declines. However, when dairy goats were exposed to increased ambient temperatures during summer months without wearing a heat blanket, RPC supplementation increased DMI (Habeeb et al., 2017). The latter finding and our present results suggest that supplementation of RPC appears to alleviate the impact of elevated ambient temperatures, enabling the animals to maintain their body temperature within the thermoneutral zone and to sustain production traits. The energy required for thermoregulatory processes is likely obtained from the higher increase in DMI of RPC cows, which, however, results in lower MY/DMI and ECM/DMI ratios in the experimental phase.

**Table 1:** Productive performance parameters (mean ± SE) of rumen-protected choline (RPC) and control (CON) groups during the pre-experimental (wk −1) and experimental (wk 1 to 4) periods^1^

Item	Pre-experimental period (wk −1), THI = 67^2^			Experimental period^3^ (wk 1 to 4), THI = 65.9^2^				
	Group		*P*-value	Group		*P*-value		
	CON (mean ± SE)	RPC (mean ± SE)	Group (G)	CON (mean ± SE)	RPC (mean ± SE)	Group (G)	Time (T)	G × T
DMI, kg/d	17.7 ± 0.61	19.3 ± 0.59	<0.01	18.6 ± 0.47	22.5 ± 0.47	<0.01	<0.01	0.68
DMI daytime,^4^ kg/9 h	7.80 ± 0.55	9.97 ± 0.59	<0.01	7.50 ± 0.20	9.55 ± 0.21	<0.01	<0.01	0.54
DMI nighttime,^5^ kg/15 h	9.90 ± 0.28	9.33 ± 0.29	0.79	11.1 ± 0.23	12.95 ± 0.24	<0.01	<0.01	0.80
WI, L/d	76.5 ± 2.12	93.9 ± 2.14	<0.01	71.6 ± 0.72	95.3 ± 1.05	<0.01	0.85	0.96
BW, kg	666 ± 4.19	669 ± 4.19	0.59	657 ± 2.38	676 ± 3.53	<0.01	0.07	0.08
DMI/BW	0.027 ± 0.001	0.029 ± 0.001	0.31	0.026 ± 0.001	0.031 ± 0.001	<0.01	0.41	0.52
BCS	3.31 ± 0.04	3.08 ± 0.03	<0.01	3.30 ± 0.02	3.12 ± 0.02	0.03	0.33	<0.01
MY, kg/d	30.3 ± 0.36	33.5 ± 0.36	0.63	30.4 ± 0.16	34.0 ± 0.16	0.76	0.35	0.99
Fat, %	3.84 ± 0.11	3.70 ± 0.10	0.27	3.84 ± 0.07	3.67 ± 0.07	0.78	<0.01	0.81
Fat yield, kg/d	1.17 ± 0.09	1.24 ± 0.08	0.88	1.16 ± 0.04	1.24 ± 0.04	0.05	<0.01	0.89
Protein, %	3.13 ± 0.09	3.15 ± 0.08	0.33	3.25 ± 0.04	3.22 ± 0.04	0.80	0.61	0.62
Protein yield, kg/d	0.95 ± 0.06	1.05 ± 0.05	0.33	0.98 ± 0.03	1.10 ± 0.03	0.16	0.60	0.93
Lactose, %	4.73 ± 0.05	4.87 ± 0.04	<0.01	4.86 ± 0.02	4.91 ± 0.02	0.13	0.10	0.85
Lactose yield, kg/d	1.43 ± 0.09	1.63 ± 0.08	0.30	1.47 ± 0.05	1.66 ± 0.05	0.56	0.24	0.99
ECM, kg/d	29.3 ± 1.88	32.1 ± 1.77	0.68	29.7 ± 0.75	32.6 ± 0.80	0.18	0.22	0.98
MY/DMI	1.67 ± 0.09	1.73 ± 0.09	0.73	1.67 ± 0.04	1.50 ± 0.04	<0.01	<0.01	0.41
ECM/DMI	1.62 ± 0.08	1.66 ± 0.09	0.68	1.62 ± 0.03	1.45 ± 0.04	<0.01	<0.01	0.23

1 All data are presented as mean ± SE, n = 9 cows per group.

2 Temperature-humidity index (THI) calculated as the daily mean of each period.

3 Baseline values of all parameters of the pre-experimental period were used as covariates.

4 Daytime = 0900 to 1800 h.

5 Nighttime = 1800 to 0900 h.

In conclusion, our results indicate that RPC supplementation reduces body surface temperatures and allow DMI, WI, BW, BCS, and milk fat yield to increase in Holstein dairy cows during warm summer days. These findings support the potential efficacy of RPC as a feeding intervention for dairy cows mitigating the adverse effects of elevated ambient temperatures. One limitation of our study, however, is that we considered the RPC feeding as a treatment for the animal in the statistical model, even though it should actually be regarded as a treatment for the pen, which in turn would restrict the degrees of freedom. Further limitations were the restricted availability of the infrared camera and technical constraints within the barn, which allowed the respiration rate measurement only once in the pre-experimental phase, and body surface temperature and rectal temperature only once during the pre-experimental and once in the experimental period. More frequent measurements of body surface and rectal temperatures are recommended to better decipher the effect of RPC on heat stress mitigation in dairy cows, particularly with the inclusion of a thermoneutral control group.
